# Shadows of the colonial past – diverging plant use in Northern Peru and Southern Ecuador

**DOI:** 10.1186/1746-4269-5-4

**Published:** 2009-02-02

**Authors:** Rainer W Bussmann, Douglas Sharon

**Affiliations:** 1 William L. Brown Center, Missouri Botanical Garden, PO Box 299, St. Louis, MO 63166-0299, USA; 2 2328 Dolphin Dr., Richmond, CA 94804, USA

## Abstract

This paper examines the traditional use of medicinal plants in Northern Peru and Southern Ecuador, with special focus on the Departments of Piura, Lambayeque, La Libertad, Cajamarca, and San Martin, and in Loja province, with special focus on the development since the early colonial period. Northern Peru represents the locus of the old Central Andean "Health Axis." The roots of traditional healing practices in this region go as far back as the Cupisnique culture early in the first millennium BC.

Northern Peru and Southern Ecuador share the same cultural context and flora but show striking differences in plant use and traditional knowledge. Two hundred fifteen plant species used for medicinal purposes in Ecuador and 510 plant species used for medicinal purposes in Peru were collected, identified,. and their vernacular names, traditional uses, and applications recorded. This number of species indicates that the healers, market vendors, and members of the public interviewed in Peru still have a very high knowledge of plants in their surroundings, which can be seen as a reflection of the knowledge of the population in general. In Ecuador much of the original plant knowledge has already been lost.

In Peru, 433 (85%) were Dicotyledons, 46 (9%) Monocotyledons, 21 (4%) Pteridophytes, and 5 (1%) Gymnosperms. Three species of *Giartina *(Algae) and one species of the Lichen genus *Siphula *were used. The families best represented were Asteraceae with 69 species, Fabaceae (35), Lamiaceae (25), and Solanaceae (21). Euphorbiaceae had 12 species, and Poaceae and Apiaceae each accounted for 11 species. In Ecuador the families best represented were Asteraceae (32 species), Euphorbiaceae, Lamiaceae, and Solanaceae (11 species each), and Apiaceae, Fabaceae, Lycopodiaceae (9 species each). One hundred eighty-two (85%) of the species used were Dicotyledons, 20 Monocotyledons (9.3%), 12 ferns (5.5%), and one unidentified lichen was used.

Most of the plants used (83%) were native to Peru and Ecuador. Fresh plants, often collected wild, were used in two thirds of all cases in Peru, but in almost 95% of the cases in Ecuador. The most common applications included the ingestion of herb decoctions or the application of plant material as poultices.

Although about 50% of the plants in use in the colonial period have disappeared from the popular pharmacopoeia, the overall number of plant species used medicinally has increased in Northern Peru, while Southern Ecuador shows a decline of plant knowledge since colonial times.

## Introduction

### Antecedents – Medicinal Plant Research and Traditional Medicine in Peru

The primary focus of this project has been the ethnobotany of medicinal plants used in Northern Peru and Southern Ecuador and the changes that have occurred since early colonial times.

Fieldwork for the present study was conducted in Southern Ecuador from 1995–2000 and in Northern Peru from 2001–2007.

Precedents for this study have been established by the late seventeenth-century plant collections of Bishop Baltasar Jaime Martínez de Compañón [1], ethnoarchaeological analysis of the psychedelic *San Pedro *cactus [2], *curandera *depictions in Moche ceramics [3], and research on the medicinal plants of Southern Ecuador [4-6] used in a field guide to the medicinal plants of the region [4,5]. Colonial records about useful plants of the region include Acosta [7], Alcedo [8], Cobo [9,10], Martínez de Compañón [11], Monardes [12], and Ruiz [13].

Considerable progress has been made in the overall taxonomic treatment of the flora of Peru over the last few decades [14]. However, while the Amazon rainforests have received a great deal of scientific attention, the mountain forests and remote highland areas are still relatively unexplored. The first floristic studies were conducted in the 1920s [15], followed by decades without any further research activity. Until the late 1990s, little work had been done on vegetation structure, ecology, and ethnobotany in the mountain forests and coastal areas of the North.

In spite of the fact that Northern Peru is the locus of what Peruvian anthropologist Lupe Camino [16] calls the "health axis," i.e., the healing center, of the old Central Andean culture area that stretched from Ecuador to Bolivia, little ethnobotanical research has been published on the rich shamanic lore found there. Early studies focused mainly of the famous "magical" and "mind altering" flora of Peru. A first study on "*cimora*" – another vernacular name for the *San Pedro *cactus (*Echinopsis pachanoi*, incorrectly identified by Cruz Sanchez as *Opuntia cylindrica*), dates back to the 1940s [17]. The first detailed study on a hallucinogen in Peru also focused on *San Pedro *and tree datura (*Brugmansia *spp.) [18-20]. A variety of works on these species followed [21-24]. Coca (*Erythroxylum coca*) also attracted early scientific attention [25-29], as did the Amazonian Ayahuasca (*Banisteriopsis caapi*) [30-33]. Chiappe et al. [34] were the first to attempt an overview on the use of hallucinogens in shamanistic practices in Peru. More comprehensive accounts were provided by Alarco de Zandra [35], Cabieses [36], and Schultes & Hofmann [37].

In his classical study of *Uña de Gato*, Peru's leading advocate for traditional medicine, and founding director of the Instituto Nacional de Medicina Tradicional del Ministerio de Salud, Fernando Cabieses [[38] p 34], points out that the Peruvian scholars Hermilio Valdizán and Angel Maldonado [15] was pioneered the study of traditional medicine, leading to the emergence of medical anthropology nearly five decades later. In the interim the botanical exploration of the Peruvian flora and medicinal plants in particular included studies by Yakovleff and Herrera [39] Weberbauer [40], Towle [41], Valdivia Ponce [42] and Brack Egg [43]. Most authors [44-50] focused on Quechua herbalism of the Cusco area. Other comprehensive studies centered on the border region of Peru and Bolivia around Lake Titicaca [51-55] and the Amazon [56-60]. Northern Peru and Southern Ecuador, in contrast, had always fallen in the shadow of the more touristically important regions, and very few studies had been conducted before the mid-nineties [61-64]. Since then our knowledge of the medicinal use of plants in the north has rapidly increased [65-70].

**Figure 1:**
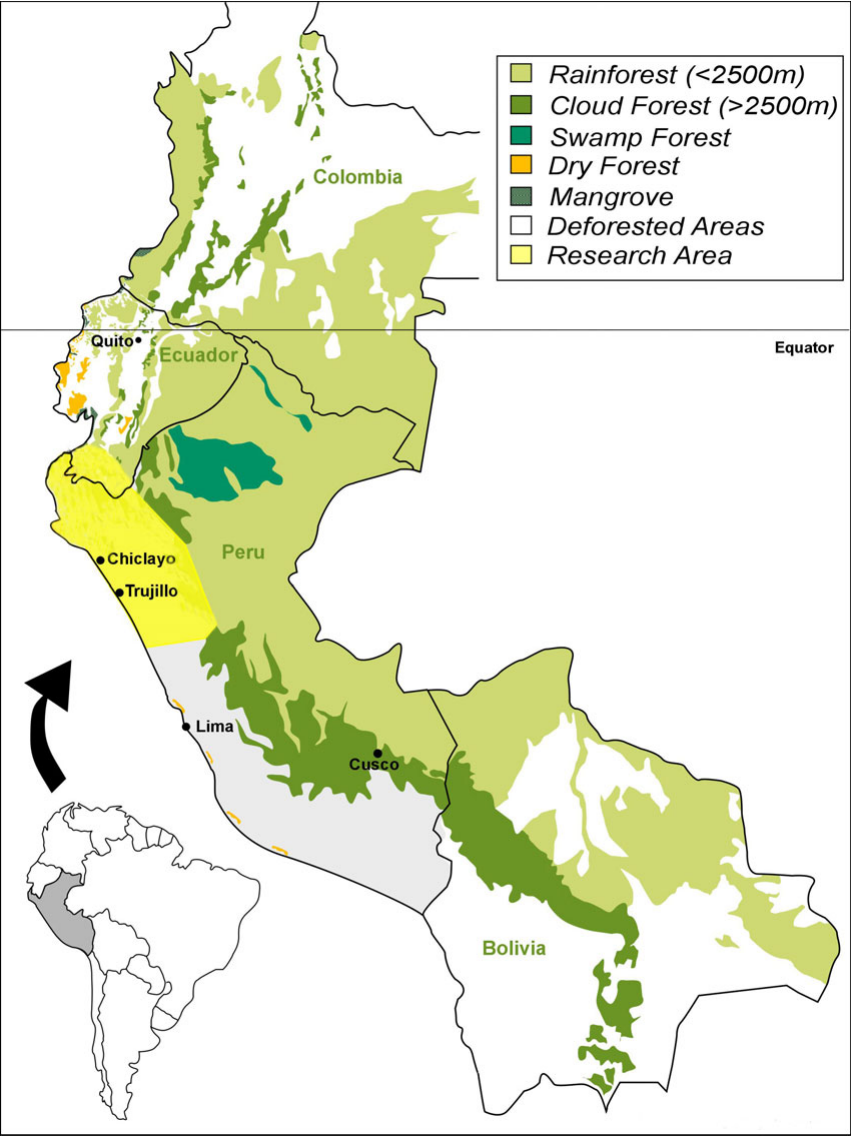
**Study area**.

**Figure 2:**
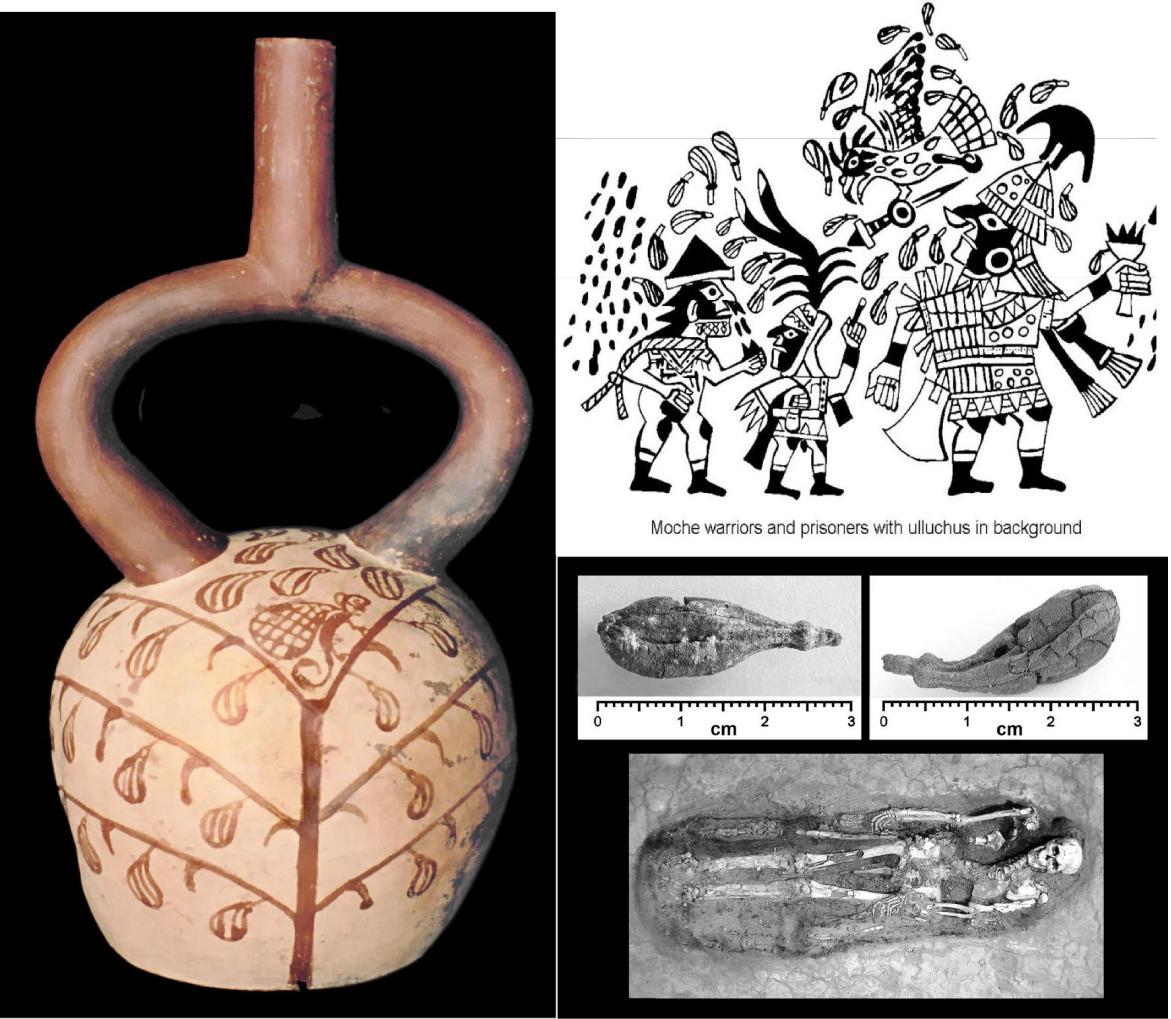
**Iconography and archaeological specimens of Ulluchu (*Guarea *sp.)**.

The traditional use of medicinal plants in this region, which encompasses in particular the Peruvian Departments of Amazonas, Piura, Lambayeque, La Libertad, Cajamarca, and San Martin, as well as the Ecuadorian Province of Loja (Fig. 1), dates as far back as the first millennium BC (north coastal Cupisnique culture) or at least to the Moche period (AC 100–800, Fig. 2), with healing scenes and healers frequently depicted in ceramics [36,71-79].

**Figure 3:**
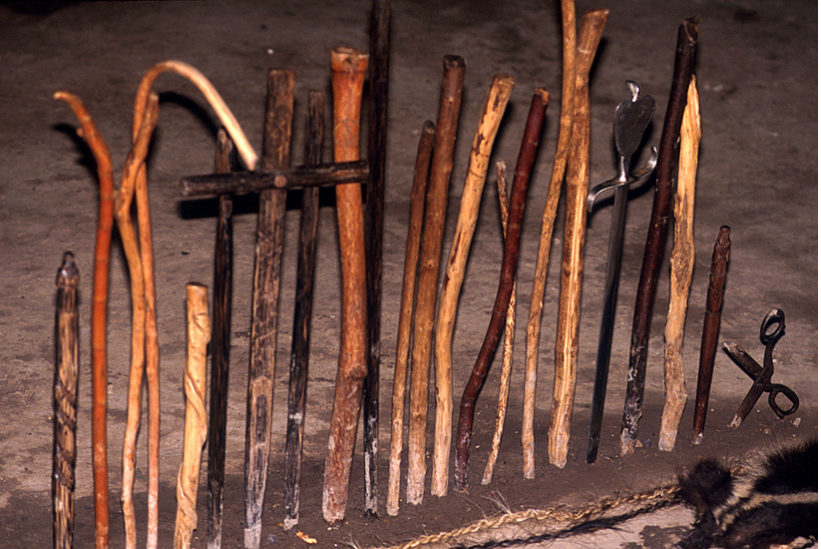
**Staffs of a Peruvian mesa**.

**Figure 4:**
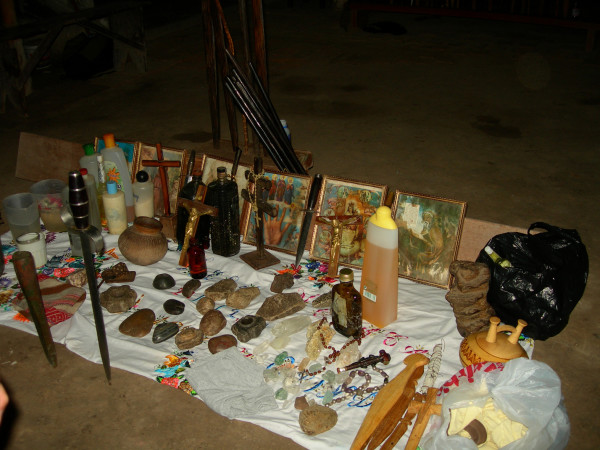
**Traditional Peruvian mesa**.

**Figure 5:**
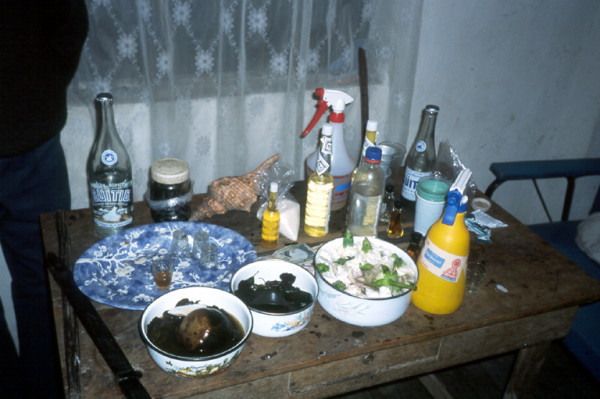
**"Westernized" Ecuadorian mesa**.

Healing altars (*mesas*) in Northern Peru often follow the old tradition by including a large variety of "power objects," frequently with a "pagan" background. Objects such as seashells, pre-Columbian ceramics, staffs, stones, etc. are very common on Peruvian *mesas *and are blended with Christian symbols such as crosses and images of saints (Figs. 3, 4 &5). Treatments are most often performed in the homes of the individual healers, who normally have their *mesas *set up in their backyards. Healers also treat patients at altars and consultation chambers (*consultorios*) in their homes, at sacred sites in the countryside, or at sacred lagoons high up in the mountains. A curing ceremony normally involves purification of the patient by orally spraying blessed and enchanted herbal extracts on the whole body to fend off evil spirits and by "Spiritual Flowerings" (*baños de florecimiento*). In most cases, the cleansing of the patients involves drinking boiled *San Pedro *juice and the nasal ingestion of tobacco juice and perfumes. Sometimes extracts of Jimson weed (*Datura ferox*), *Brugmansia *spp., and tobacco are also used to purify the patients. While the incantations used by healers during their curing sessions include Christian components (e.g., the invocation of Christ, the Virgin Mary, and any number of saints), references to Andean cosmology (e.g., to the *apus *or the spirits of the mountains) are very common. The use of guinea pigs as diagnostic instruments is standard in Northern Peru [2,80-83].

## Methods

### Plant Collections

Plants in Peru were collected in the field, in markets, and at the homes of traditional healers (*curanderos*) visited in August–September 2001, July–August 2002, July–August 2003, June–August 2004, July–August 2005, July–August 2006, June–August 2007 and June–August 2008. The specimens are registered under the collection series "RBU/PL," "ISA," "GER," "JULS," "EHCHL," "VFCHL," "TRUBH," and "TRUVANERICA," depending on the year of fieldwork and collection location.

Vouchers of all specimens were deposited at the Herbario Truxillensis (HUT, Universidad Nacional de Trujillo) and Herbario Antenor Orrego (HAO, Universidad Privada Antenor Orrego Trujillo). In order to recognize Peru's rights under the Convention on Biological Diversity, especially with regard to the conservation of genetic resources in the framework of a study treating medicinal plants, the identification of the plant material was conducted entirely in Peru. No plant material was exported in any form whatsoever.

The majority of plants in Ecuador were collected during field visits in August–September 1995, May 1996, August–November 1996, March 1997, and June–July 1997. The specimens were registered under the collection series "Bejar" and "CORD."

Vouchers of all specimens collected in Ecuador were deposited at the Herbario Estación Científica San Francisco (ECSF), Herbario Loja (LOJA), Herbario Nacional de Ecuador (QCNE), and Herbario de la Pontificia Universidad Católica de Ecuador (QCA). The identification of the plant material was conducted entirely in Ecuador. No plant material was exported in any form whatsoever.

For the surveys of traditional plant uses, detailed questionnaires were used; these included questions about plant origin, vernacular name, illness category, recipe formulation, pricing, and quantities sold. The authors decided to maintain traditional illness categories given by the informants, rather than trying to convert these categories to fit the western biomedical system. Surveys were conducted in Spanish by fluent speakers. Surveyors would approach healers and market vendors and explain the premise for the study, including the goal of conservation of medicinal plants in the area. The same number of healers (both male and female) was interviewed in Ecuador and Peru, after explaining the scope of the study and obtaining prior informed consent. While the Peruvian healers interviewed all mostly delighted to have their names given in the acknowledgments, their Ecuadorian colleagues strongly insisted that their identities should not be revealed. In both countries, the healers did not want to be listed as co-authors.

In contrast to Peru, no medicinal plants were sold in markets in Ecuador, indicating a large difference in the availability of plants. For this reason, market surveys concentrated on Peru only. In the main markets in Trujillo and Chiclayo, all vendors (110) were asked to participate in the surveys, but due to expected resistance information could not collected from everyone. From those who gave their prior informed consent, information was collected regarding their inventory of medicinal plants. However, all vendors agreed to sell their plants to the surveyors, even when not giving any use information. For this reason, the market species inventory was complete. The vendors were also asked to list the ten most commonly sold plants, and ten plants that were disappearing from the market. Of the plants that were most commonly sold and declining, information was also collected on the location (*montaña *– mountain forest, *costa *– coast, *sierra *– highlands, or *selva *– jungle), origin (*pueblo *– village), cost per unit sold (*soles*), units sold per week (*bultos *– bundles, *paquetes *– packets), the time when the vendors' suppliers distribute goods, and any other information concerning the popularity of the plant. At each market, the number of medicinal plant vendors was counted to estimate how the vendors who participated in the study were representative for the entire market.

### Nomenclature

The floras of North Peru and Southern Ecuador are part of the same floristic region and overlap to a very large extent. Both areas harbor about 4,000 species of vascular plants. The nomenclature of plant families, genera, and species follows the *Catalogue of the Flowering Plants and Gymnosperms of Peru *[14] and the *Catalogue of Vascular Plants of Ecuador *[84]. The nomenclature was compared to the TROPICOS database. Species were identified using the available volumes of the *Flora of Peru *[85], as well as Jørgensen & Ulloa Ulloa [86], Pestalozzi [87] and Ulloa Ulloa & Jørgensen [88], and the available volumes of the *Flora of Ecuador *[89], and reference material in the herbaria HUT, HAO, QCA, LOJA, and QCNE.

### Cluster analysis of plant records

The goal of cluster analysis is to group objects together that are similar. Data in the literature and market collections were organized in an Excel spreadsheet that arranged species in rows and sources in columns. Individual cells contained qualitative presence/absence data, represented by numerical values "1" or "0." The Excel spreadsheet was imported into NTSYSpc (version 2.10L) and a (dis)similarity matrix was produced using the Simple Matching Coefficient that measures the degree of similarity/dissimilarity between all pairs of markets. Next, a dendrogram (tree) was generated with the UPGMA-SAHN method. Since a cluster analysis will always yield clusters, it is necessary to demonstrate how well the analysis represents the original (dis)similarity matrix. To this end, the tree matrix was transformed into a matrix of ultrametric distances and the latter matrix was statistically compared with the original (dis)similarity matrix. The resulting correlation coefficient "r" between both matrices (normalized Mantel statistic Z) can be used as a measure of goodness of fit for cluster analysis. The degree of fit can be interpreted subjectively as follows: 0.9 ≤ r: very good fit; 0.8 ≤ r < 0.9: good fit; 0.7≤ r < 0.8: poor fit; r < 0.7: very poor fit.

## Results and discussion

### Indigenous Nomenclature

The healers interviewed belonged entirely to the Mestizo community (people of mixed Indigenous and European descent). The naming of plant species follows three general patterns. Plant names already used by original indigenous populations are often maintained, although slightly modified. Plants similar to species already known, or with similar habitus, often receive the same name (transposition). In other cases, completely new names are created (neology) [90].

The vernacular names of the plants used in Northern Peru reflect the historical development of plant use in the region. Introduced species (e.g. *Apium graveolens *– Apio, *Foeniculum vulgare *– Hinojo), native species similar to species found in Spain (e.g. *Adiantum concinnum *– Culantrillo, *Matricaria frigidum *– Manzanilla), as well as species growing mostly in the coastal regions of the area (e.g. *Alternanthera porrigens *– Sanguinaria), are often addressed with names derived from Spanish roots. Plants from the mountain forests, and especially the Andean highlands or the Amazon, are often known by their Quechua names (e.g. *Pellaea ternifolia *– Cuti Cuti, *Amaranthus caudatus *– Quihuicha, *Banisteriopsis caapi *– Ayahuasca). A few plant names can be traced back to Mochica (the original indigenous language spoken at the coast of Northern Peru) roots (e.g., *Nectandra *spp. – Espingo). Van den Eynden et al. [90] observed similar patterns in Southern Ecuador, although her study focused only on edible species. Nine hundred thirty-eight vernacular names were recorded for 510 plant species. About one third of all names represented Quechua names or had Mochica roots, while 66.5% of all names were of Spanish origin or at least had Spanish components. In comparison, 41% of the vernacular names of edible plants in Southern Ecuador were found to be of Spanish origin. More than half of the indigenous species carried only one vernacular name, with the remaining species carrying a variety of indigenous names, often derived from the same root. In comparison, almost 75% of the introductions were known by one name only. The slight differences in plant names indicate that the species have been used in the region for a long time and that their names reflect small variations in the local dialects.

### Plant Uses

A total of 510 taxa belonging to 250 genera and 126 families are now on record for Peru. Of these, 504 could be identified, most of them to the species level.

Four hundred thirty-three species (85%) were Dicotyledons, 46 (9%) Monocotyledons, 21 (4%) Pteridophytes, and 5 (1%) Gymnosperms. Three species of *Giartina *(Algae) and one species of the Lichen genus *Siphula *were used. Four hundred twenty-two species (83%) were indigenous to Northern Peru, while 87 species (17%) were introductions. Many of the introduced species were medicinal plants that were brought in for the treatment of European diseases during colonial times (Table 1). The families best represented were Asteraceae with 69 species, Fabaceae (35), Lamiaceae (25), and Solanaceae (21). Euphorbiaceae had 12 species, and Poaceae and Apiaceae each accounted for 11 species.

**Table 1:** Main plant groups used in Northern Peru and Southern Ecuador and plant origin

	**Number of species PERU**		**Number of species ECUADOR**	
		%		%
**Dicotyledoneae**	434	85	182	85
**Monocotyledoneae**	46	9	20	9.3
**Pteridophyta**	21	4	12	5.5
**Gymnospermae**	5	1	0	0
**Algae**	3	0.7	0	0
**Lichenes**	1	0.3	1	0.2
**Total**	**510**	**100**	**215**	**100**
				
**Indigenous**	**424**	**83**	**179**	**83**
**Introduced**	**86**	**17**	**36**	**17**

A total of 215 taxa belonging to 158 genera and 76 families are on record for Ecuador. Of these, 214 could be identified, most of them to the species level. The number of plants in use represented only a fraction (about 5%) of the flora of the region. The families best represented are Asteraceae with 32 species, Euphorbiaceae, Lamiaceae and Solanaceae (11 species each), and Apiaceae, Fabaceae, and Lycopodiaceae (9 species each). One hundred eighty-two (85%) of the species used were Dicotyledons, 20 Monocotyledons (9.3%), 12 ferns (5.5%), and one unidentified lichen was used. One hundred seventy-nine species (83%) were indigenous to Southern Ecuador, while 36 species (17%) were introductions (Table 1). Many of the introduced species are medicinal plants brought in during colonial times.

Five hundred ten plants with medicinal properties were registered in Northern Peru. The same species was often used for various medical conditions and applied in different ways for the same condition. For example, nervous disorders might be treated using different parts of a plant in different applications, e.g., topical (as a poultice or bath), oral (ingestion of plant extracts), and in the form of a "*seguro*," a bottle with herbs and perfumes that serves as a protecting charm. Two thousand four hundred ninety-nine different uses were registered for the 510 species encountered. Two hundred seventy-eight different medical conditions were recorded. Most plants were used for the treatment of multiple ailments.

The highest number of species (207, 40.4%) were used for the treatment of "magical/ritual" ailments like *mal aire *(bad air; illness caused by spirits who influence passing adults), *mal viento *(bad wind, similar to *mal aire *but affecting mostly children), *susto *and *espanto *(fright, caused by a shock event in one's life or environment), *mal ojo *(evil eye, an illness mainly in children caused by persons with a negative glance), and *envidia *(envy, an illness of adults caused by the envy of other persons). Ninety-eight species (19.1%) were used to treat psychosomatic and nervous system problems, while respiratory problems were treated with 95 species (18.5%). Kidney and urinary tract disorders were treated with 85 species (16.6%), rheumatic and arthritic symptoms with 45 species (8.8%), and infections of female organs were treated with 66 species (12.9%).

Two hundred fifteen plants registered in Southern Ecuador had medicinal properties. The highest number of species (39) were used for the treatment of "magical" (psychosomatic) ailments. Fever/Malaria (25 species), respiratory disorders (34 species), rheumatism (23 species), and nervous system problems (20 species) followed.

Most treatments were performed in the homes of the individual healers, who normally had their *mesas *(healing altars) already set up. In most cases in Southern Ecuador, a "Western" altar with few power objects was employed (Fig. 5), in contrast to Northern Peru, where normally a "traditional" *mesa *was set up (Figs. 3, 4). This difference is rooted in the fact that traditional healing was illegal in Ecuador until the constitutional revision of 1998. Additionally, traditional cures are often performed outdoors, either close to sacred lagoons or waterfalls or at special ceremonial sites. A curing ceremony normally involves purifications of the patient by orally spraying blessed and enchanted herbal extracts on the whole body to fend off evil spirits.

### Magical/Ritual healing

*Mal aire *(bad air), *mal viento *(bad wind), *susto *and *espanto *(fright), *mal ojo *(evil eye) and *envidia *(envy) are seen as very common illnesses in Andean society. Causes include sudden changes in body temperature, any kind of shock, spells cast by other people, poisoned food, etc. Medicinal problems caused by outside influences were reported in a wide variety of studies [51,90]. The Western concept of "psychosomatic disorders" comes closest to characterizing such illnesses. These illness categories are deeply rooted in Andean society, and Western medicine does not offer efficient alternatives to traditional treatment. This might explain why this category has still such outstanding importance.

Two hundred seven plant species (40.4% of all species encountered) were named for treating these disorders. In addition, seven species (1.4%) were hallucinogens used in curing ceremonies.

Treatment in many cases involved the participation of the patient in a cleansing ceremony or *limpia*. This could either be a relatively simple spraying with perfumes and holy water, or an all-night ceremony involving the healer's curing altar (*mesa*). In the days after an all-night ritual, patients are normally treated with a *florecimiento *(flowering bath) in order to relieve them of any remaining adverse symptoms or spirits. In addition, patients frequently receive *seguros *(herbal amulets) for protection against further evil influences and for good luck. *Seguros *are flasks filled with powerful herbs, as well as perfumes, laminated saint images, and the hair and fingernails of the patient.

The enormous number of plant species used for the treatment of psychosomatic disorders indicates that the *curanderos *of Northern Peru are valued specialists who are consulted mainly for these conditions. This is all the more interesting since Western medicine has still not found efficient treatments for psychosomatic disorders. The plant species used for "magical/ritual" disorders come mostly from the high Andes, especially from the vicinity of sacred lakes, since plants from those regions are regarded as especially powerful. This links the present day curing practices directly to ancient Andean cosmology [16]. The use of purgatives and laxatives to literally "expel" evil spirits is also very common.

In contrast, in Ecuador only 59 applications fell into the magical category, with 39 plant species named to treat these disorders. *Mal aire*, *susto*, and *sorcery *were the most common magical illnesses encountered. Treatment in many cases involved the participation of the patient in a cleansing ceremony (*limpia*). This could either be a relatively simple spraying with perfumes and holy water, or an all- night ceremony involving the healer's curing altar (*mesa*). In addition, patients frequently received *seguros *(herbal amulets) for protection against further evil influences and for good luck.

### Nerves and Psychosomatic Problems

The enormous role that *curanderos *play in the area of treatment of psychosomatic and nervous system problems becomes even more apparent when considering that a total of 98 species (19.1%) involved the treatment of nervous system disorders like depression, anxiety, insomnia, etc. Some of the plants used (e.g., *Valeriana *spp.), are used worldwide for the treatment of nervous disorders.

A fair number of species (20) were used to treat nervous system disorders in Ecuador. This includes general nervous disorders, depression as well as psychological fatigue.

### Respiratory System

Respiratory system problems, like the common cold, flu, bronchitis, and asthma represented the most common bodily illnesses treated by healers in Northern Peru. The often damp conditions in local homes, leading to high mold counts as well as insufficient air circulation, account for the prevalence of these ailments. Many houses in rural areas have open stoves, with smoke causing constant irritation to the pulmonary system. *Curanderos *use 95 plant species (18.5%) for respiratory problems.

In many rural areas in Ecuador and Peru, the smoke of cooking stoves still escapes through the roof or doorway. Consequently, a large variety of respiratory problems is very common. Houses are also often very damp and cold, especially at higher altitudes. This leads to a high incidence of respiratory infections. Forty-five applications included respiratory ailments, with 34 plant species employed to treat respiratory conditions. The most prevalent respiratory problems were the common cold, cough, flu, and bronchitis.

### Urinary System (Kidneys, Bladder)

Disorders of the urinary system included kidney and bladder infections and kidney stones. Altogether 85 plant species used (16.6%) focused on the urinary system. Some of the species employed (e.g., *Chanca Piedra*, literally "Stonecrusher" (*Phyllanthus *spp.),) have already entered the international market.

### Rheumatic Problems

The housing conditions already described, as well as difficult working conditions, led to a wide spectrum of muscular-skeletal disorders, including rheumatism, arthritis, bone and muscle pain. Forty-five species (8.8%) used fell into this illness category. Treatment involved the application of a poultice to the affected body part. Willow (*Salix *sp.), well known for its content of acetacetylic acid, was used orally as an analgesic.

Twenty-eight applications were for rheumatic problems, with 23 plant species used to treat rheumatic and musculo-skeletal ailments in Ecuador. Most of these arose from the living conditions of the population, mainly damp and cold caused by insufficient insulation, heating, and poor circulation in rural houses. Rheumatic conditions included arthritis, rheumatic fevers, muscular and skeletal pains, as well as body and joint pain.

### Internal Organs (Liver, Gallbladder, Diarrhea, Colic)

In Peru, internal organ disorders fell far behind the most commonly treated medical conditions. This is another indication that *curanderos *in Northern Peru are to a large extent specializing in the treatment of psychosomatic disorders, and that "bodily" illnesses are treated more as a sideline. Internal organ problems treated included: liver problems (61 species, 11.9%); stomach problems, including ulcers (51 species, 9.9%); colic (59 species, 11.5%); digestive tract inflammations (33 species, 6.4%), diarrhea (17 species, 3.3%), and gallbladder problems, including stones (18 species, 3.5%). The cleansing of the digestive system through enemas (4 species, 0.8%) and by employing laxatives/purgatives (19 species, 3.7%) was also observed.

In Ecuador, the highest number of species was used to treat internal organ and digestive system disorders. This included mostly urinary tract and kidney infections (28 species, 13% of all species used), liver problems (19 species) and stomach ailments, including ulcers (23 species). Eighteen species were used for the treatment of diarrhea and colic.

### Gynecological Problems

Gynecological problems were among the most important medical conditions treated by *curanderos*, independent of the gender of the healer. Infections of the ovaries, uterus, and vagina as well as post partum infections were very common conditions for which women sought the help of healers. Infections of this kind involved 66 species (12.9% of the total). Furthermore, 25 species (4.9%) involved facilitation of childbirth, such as easing of dilation. The same species were often used to ease menstrual cramps and to regulate the menstrual cycle. Birth control, female fertility, and abortion were treated with only 8 different species (1.6%), only one of which (*Ruta graveolens*) was used to induce abortions.

Menstruation problems and complications in childbirth were very common medical conditions in Southern Ecuador. Sixteen plants were employed to treat these disorders, with six species used to cure vaginal infections, and four species each for the treatment of childbirth complications and menstrual regulation.

### Skin Problems

Skin infections, either fungal or bacterial, as well as sunspots, moles, pockmarks, and malnutrition blemishes were frequently observed in Northern Peru. Traditional healers were consequently consulted to treat these conditions. Forty species (7.8%) were used. Fungal infections are particularly difficult to treat using Western medicine, and the use of plants to alleviate such infections is thus of particular interest.

### Heart and Circulatory System

Traditional healers were frequently consulted to treat heart problems and disorders of the circulatory system. Typical heart conditions, including heart pain involved 44 species (8.6%). Blood pressure issues were rather insignificant, with high blood pressure treated with seven species (1.4%), and low blood pressure with three species (0.6%). Interestingly, *Erodium cicutarium *was used to treat both conditions. Most treatments of the circulatory system involved the purification of the blood in order to improve the general condition of the patient. Forty-four species (8.6%) were used for blood purifications.

The main application for circulatory system problems in Ecuador was the treatment of heart pain. Twelve species were used for the treatment of heart conditions, including heart attacks and heart pain. Three species were used to regulate hypertension, and one species helped to lower cholesterol levels.

### Weight Management/Cholesterol

The fashionable concept of "weight management" and conditions relating to obesity has entered the domain of Peruvian healers. Diabetes, especially in overweight patients, occurred as a prominent medical condition with 33 species (6.4%) used for treatment. The high incidence of diabetic conditions seems to point towards a change in lifestyle and nutrition among the local population. All healers readily acknowledged the negative influence of high cholesterol levels, and 11 plant species (2.1%) were used specifically to lower cholesterol. Fifteen species (2.9%) involved weight loss therapies, while plants used for weight gain were insignificant (two species, 0.4%).

### Inflammation

Fifty-nine plant species (11.5%) were used to treat general inflammation of the body. In addition, throat and tonsil infections were treated with seven species (1.4%).

In Ecuador inflammations were treated with 20 plant species.

### Wounds and Hemorrhages

Wound infections and bleeding resulting from accidents are very common in the Northern Peruvian work environment and are a major concern, especially in rural areas with 8.4% of all plants (43 species) used for the treatment of wounds. An additional 12 species (2.3%) were used for treatment of bleeding and hemorrhages.

### Bones

The treatment of fractures, sprains and the like were cured with 13 species (2.5%).

### Male Problems (Impotence, Prostate, Hair loss)

Typical male problems, like prostate inflammations and disorders, impotence, and hair loss, had a relatively prominent role in the treatments observed. Twenty-three species (4.5%) used involved prostate inflammations and problems in urinating. "Hair loss" was treated with 17 species (3.3%). Finally, 12 species (2.3%) focused on the treatment of male impotence, on the improvement of potency, or the plants were simply used as aphrodisiacs.

### Fever

"Fever" included a variety of conditions, from fevers accompanying flu, to fever as a result of malaria. Seventeen plant species (3.3%) were used. Malaria was recognized as a parasitic infection and treated accordingly, while other plant species were used to treat fever as a symptom, mainly focusing on lowering body temperature.

Yellow fever is very rare in Southern Ecuador. The frequent occurrence of the term in local illness categories is thus somewhat surprising. Malaria is rather common in some parts of Loja province and does indeed represent a serious threat to the population, especially during the rainy season. Therefore, it is not surprising that 25 plants were used to treat this condition.

### Cancer and Tumors

Various cancers and tumorous conditions were also treated by *curanderos*. Treatment of such cases often involved a single species at a time, with a total of 22 plant species (4.3%) used. The use of plant species in this field could provide particularly interesting leads in medicinal development.

### Infection (Bacterial and Viral, Parasites)

Infections caused by bacteria, viruses, and various parasites are common in many developing countries. Bacterial infections treated included cholera, tuberculosis, and gangrene (11 species, 2.1%), while viral infections were mostly related to dengue fever, yellow fever and measles (seven species, 1.4%). Intestinal, urinary tract, and female organ infections have already been mentioned.

Parasites like amoebas, plasmodia, and worms were treated with 11 different species (2.1%).

Bacterial and viral infections, especially wounds, were a major concern in Ecuador. Twelve plant species were used, mostly as poultices, to treat infected wounds, one of them to treat gangrene. Eleven species were used to treat internal bacterial infections.

### Pain

Five species (1%) involved the treatment of general pain, intense body pain (e.g., caused by dengue fever), as well as tooth pain and the follow-up after extraction.

Thirteen species were used as analgesics, especially for the treatment of headaches, general pain, and toothache in Ecuador.

### Brain

Memory loss and confusion, caused by old age were treated with six plant species (1.2%).

### Other Uses

Rare disorders treated included: contusion and hangover (three species, 0.6%); animal bites (snake bites, rabies) (five species, 1%); eye problems (nine species, 1.8%); cysts (six species, 1.2%); headache (six species, 1.4%); bad breath and detoxification (drug and alcohol abuse); hemorrhoids (four species, 0.8% each); paralysis (one species, 0.2%); anemia, ear and hearing problems, internal bleeding and varicose veins (three species, 0.6% each); alertness and nosebleeds (two species, 0.4% each); abscesses, anesthetics, anal and vaginal pimples, antiseptics, cramps, mouth bitterness, sarna, and waking a person who has fainted (one species, 0.2%).

In Southern Ecuador, a wide variety of plants were used to treat other ailments: eEight species served as purgatives, five were used to remedy cramps. Hemorrhages, eye infections, skin disorders, and parasites (amoebas and worms) were treated with four species each.

Three species were used for blood purification, diabetes, and cancer. Insect bites (2), hernia, fractures, allergies, leucorrhea and venereal disease (one species each) were less important medical conditions treated.

#### Parts of Medicinal Plants Used and Modes of Application

**Figure 6:**
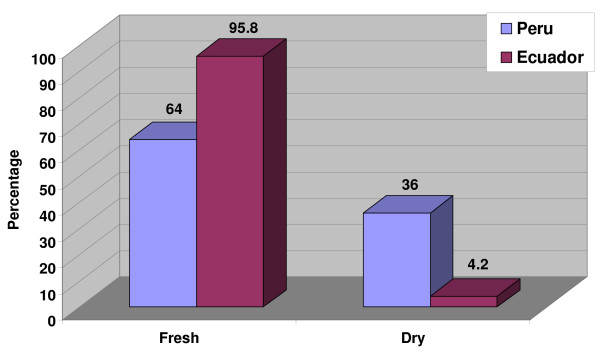
**Condition of medicinal plants used in the study area**.

Almost two-thirds (64%) of the remedies employed in Northern Peru are prepared using fresh plant material. Many of the introduced species are cultivated in fields and gardens, but the majority of the indigenous species are collected wild. This indicates that a widespread system of plant collectors is needed to supply the fresh plant material needed in traditional medicine. Most healers agreed that in most cases dried material could be used if fresh plants were not available. In 36% of all cases the remedies were prepared using specifically dried plant material. The main explanation for this, however, was that the plant material had to be transported from other regions, and thus fresh material was not available (Table 2, Fig. 6).

**Table 2:** Plant constitution

**Constitution**	**Number of uses PERU**		**Number of uses ECUADOR**	
		%		%
**Fresh**	**626**	**64**	**207**	**95.8**
**Dry**	**355**	**36**	9	4.2
**TOTAL**	**981**	**100**	**216**	**100**

**Figure 7:**
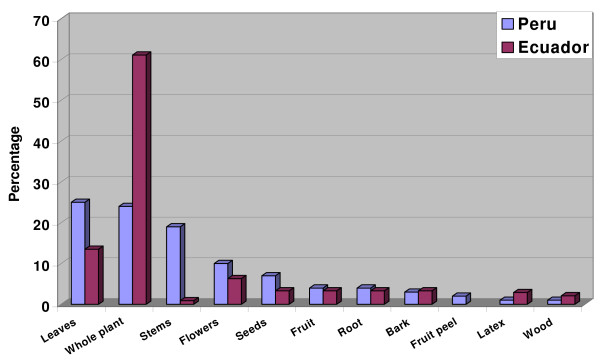
**Parts of medicinal plants utilized in Ecuador and Peru**.

Northern Peruvian *curanderos *prefer to use either the leaves (in 25% of all uses) or the whole plant (24%) for the preparation of their remedies. In 19% of all cases, the stems of the plants were used, most commonly together with the leaves. Flowers (10%), seeds (7%), fruits and roots (4% each), bark (3%), fruit peel (2%), and latex and wood (1% each) were only used for a small number of preparations (Table 3, Fig. 7).

**Table 3:** Plant part used for medicinal purposes

**Plant Part**	**Number of uses PERU**		**Number of uses ECUADOR**	
		**%**		**%**
**Leaves**	191	25	32	13.5
**Whole plant**	**184**	**24**	**146**	**61.1**
**Stems**	146	19	2	0.9
**Flowers**	73	10	15	6.3
**Seeds**	55	7	8	3.3
**Fruit**	31	4	8	3.3
**Root**	28	4	8	3.3
**Bark**	20	3	8	3.3
**Fruit peel**	12	2		
**Latex**	9	1	7	2.9
**Wood**	6	1	5	2.1
	**755**	**100**	**239**	**100**

Healers in Northern Peru often employ very sophisticated mixtures of a variety of plants in their treatments. The use of single species was rare. Most commonly, plant material was boiled in water, or in some cases in sugarcane alcohol (*aguardiente*), to extract the active compounds. In some cases, plant material was macerated in cane alcohol or wine for longer periods of time before use.

The *curanderos *all had strikingly exact recipes for treatment, with very specific quantities of plant material used to prepare remedies. These quantities did not differ greatly from one healer to another. Simultaneously, the amount of a specific remedy that was given to a patient was very similar among the different *curanderos*.

Almost all remedies were prepared from fresh plant material (96%) (Table 2, Fig. 6). All of the introduced plant species were cultivated in fields and gardens, while most of the indigenous species were collected in the wild.

**Figure 8:**
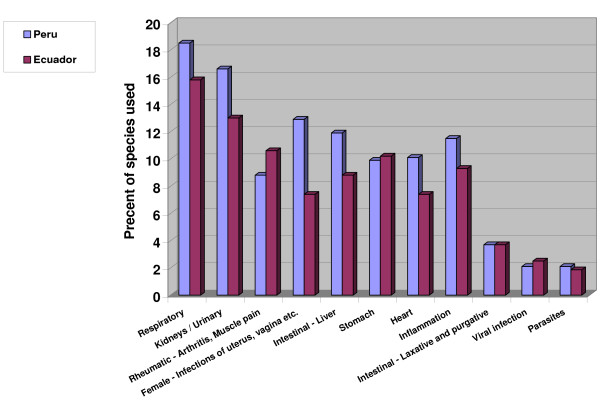
**Plant use percentage for treatment of various illness concepts in Ecuador and Peru I**.

The most frequent way to administer remedies was to prepare a decoction and ingest it orally (52% of all uses), followed by application as a poultice (38%, plant crushed or boiled and applied). Seven percent of all plant uses entailed the preparation of a *seguro*, a bottle or small flask filled with plant material along with various perfumes. This amulet has to be carried by the patient at all times, or it is placed in the house and used for periodic blessings. *Seguros *contained anything from a handful to more than three-dozen different ingredients. In two percent of the plant uses the material was employed to fabricate charms, and in one percent of all applications the plant material was burned as incense, with the smoke inhaled for treatment (Table 4, Fig. 8).

**Table 4:** Preparation and application methods for medicinal plants:

**Application**	**Number of uses PERU**		**Number of uses ECUADOR**	
		**%**		**%**
**Oral**	429	52	168	67.8
**Topical**	315	38	79	31.8
**Seguro**	**60**	**7**	**0**	**0**
**Charm**	**14**	**2**	**0**	**0**
**Incense**	**10**	**1**	**1**	**0.4**
				
**TOTAL**	**828**	**100**	**248**	**100**

A completely different picture emerges in Ecuador. In most cases (61%) the whole plant was used for medicinal purposes, followed by leaves (13%), and flowers (6%); the seeds, roots, bark, fruits, and latex were rarely used (3% each) (Table 3, Fig. 7). This indicates that Peruvian healers have a much more sophisticated knowledge of plant properties than their Ecuadorian colleagues.

**Figure 9:**
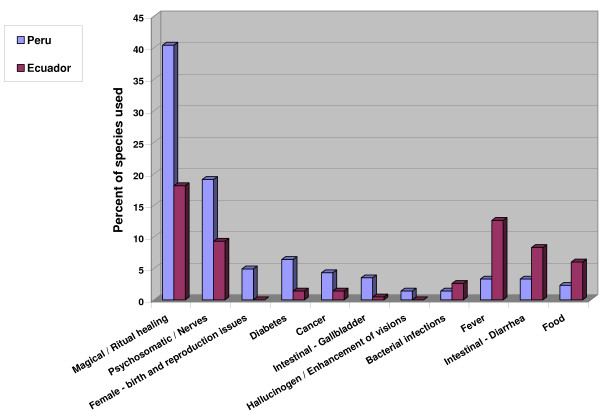
**Plant use percentage for treatment of various illness concepts in Ecuador and Peru II**.

In both countries, diseases and other health problems were most frequently treated with decoctions of various plant species. Of all preparations mentioned, plants were mostly boiled in water or sugarcane alcohol (*aguardiente*). However, profound differences exist in the type of diseases treated. The healers of both regions used a similar percentage of their pharmacopoeia for the treatment of "common" diseases, like respiratory disorders, kidney and urinary infections, rheumatic disorders, infections of female organs, heart problems, intestinal infections, parasites etc. (Fig. 8). In contrast, hardly any plants were used in Ecuador for the treatment of psychosomatic disorders, for ritual healing purposes, reproductive issues, diabetes, cancer, gallbladder problems, and as hallucinogens. All these uses were very common in Peru where, in fact over 40% of all plant species used had some function in ritual treatments. In contrast, Ecuadorian healers used a higher percentage of their plants to treat fevers and diarrhea (Fig. 9.)

**Figure 10:**
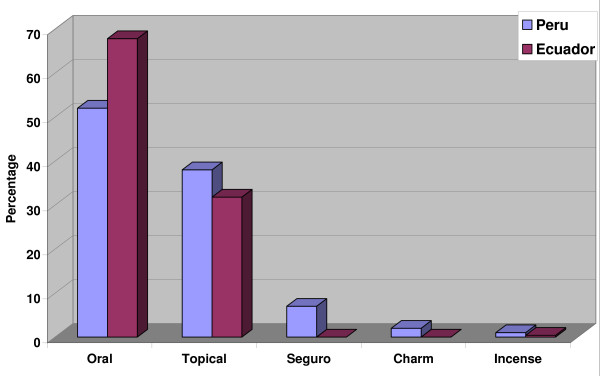
**Medicinal plant preparation in Ecuador and Peru**.

The most frequent way to administer remedies was as to prepare a decoction and ingest it orally (67.8%), followed by application as a poultice (31.8%, plant crushed or boiled and applied). Only 0.4% of the plants were burned for inhalation (Table 4, Fig. 10).

#### Food and Spices

A variety of species normally used as food also had some medicinal applications, mostly as nutritional supplements to treat mineral and vitamin deficiencies and malnutrition, and were prepared and served as side-dishes or as ingredients of normal meals. Old Andean crops like Quinoa (*Chenopodium quinoa*), Kwicha (*Amaranthus caudatus*), Tarhui (*Lupinus mutabilis*) and Maca (*Lepidium meyenii*) – now globally used as a supplement – featured most prominently. Coastal species like Algarrobo (*Prosopis pallida*) were also used. Altogether 12 species (2.3%) were used in this way.

Ecuadorian healers used 13 plant species, predominantly European introductions like *Pimpinella anisum, Foeniculum vulgare, Origanum vulgare*, etc. as food and spices, in addition to their medicinal uses.

#### Ceremonial

Palm staffs (*Bactris *spp.) continue to be used as power objects on both Northern Peruvian and Ecuadorian *mesas*.

#### The pharmacopoeae of Southern Ecuador and Northern Peru – Colonial regimes and their influence on plant use

**Figure 11:**
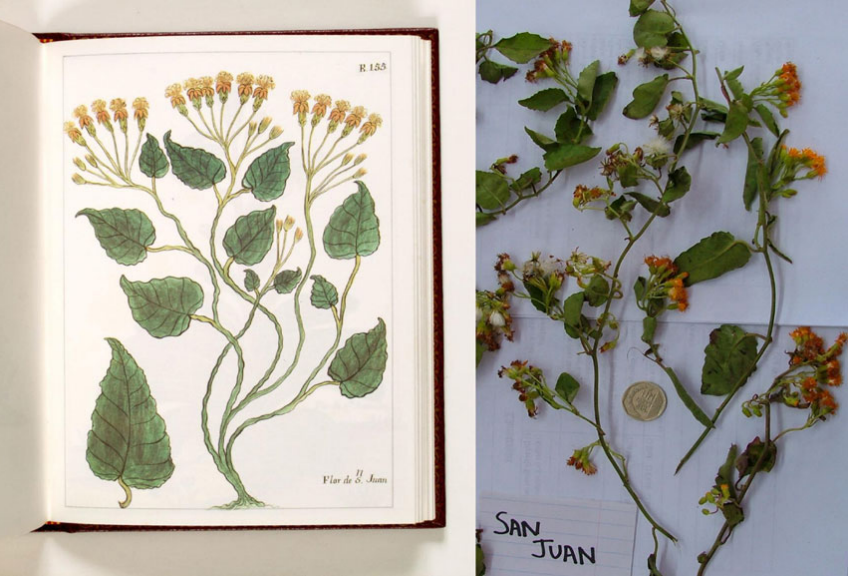
**"San Juan" in colonial iconography and market specimen**.

**Figure 12:**
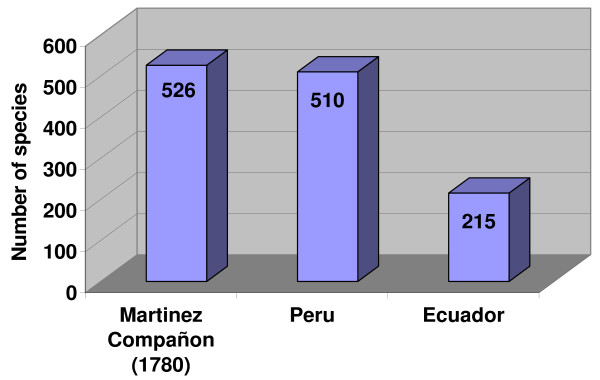
**Number of plant species used in late colonial times and 2008**.

**Figure 13:**
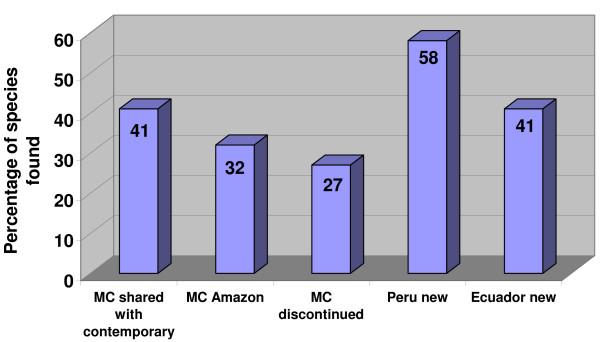
**Change in plant portfolio from colonial times to 2008**.

The differences in medicinal plant use between Southern Ecuador and Northern Peru are striking. Both regions share the same cultural background and have a very similar flora, with a comparable number of plant species that to a large extent overlap. However, the medicinal flora of Southern Ecuador includes only 40% of the species used in Northern Peru. The differences in traditional medicinal use can be explained by comparing the history of the pharmacopoeiae of both areas from the start of the colonial period until today. Colonial chroniclers often included detailed descriptions of useful plants in their reports. The most comprehensive early accounts of the economically interesting flora of Northern Peru and Southern Ecuador were provided by Monardes [12], Acosta [7], and Cobo [9,10]. Later treatments were included in Alcedo [8]. Martínez Compañon, Archbishop of Trujillo, had a complete inventory of his dioceses prepared [11]. Finally, Ruiz and Pavón provided the first real botanical inventory of the region [13]. The account of Martínez Compañon [11] provides the best baseline for a comparison of the colonial and modern medicinal flora of the region. The work includes detailed paintings for every species, which allows a close comparison with the modern medicinal flora, indicating that the vernacular names of useful plants have not changed significantly since colonial times (Fig. 11). It contains 526 useful plant species. A preliminary review of this work seems to indicate that the number of plants used has not changed significantly since the late 1700's, with over 500 plant species still found in modern Peruvian markets (Fig. 12). A closer comparison shows, however, that only 41% of the species mentioned by Martinez Compañon [11] are still sold nowadays in Peru. An additional 32% are still used in the Amazon basin, but do not reach the coastal markets anymore. Twenty-seven percent have completely disappeared from modern day use. This means that 58% of the species sold in Peruvian markets and 41% of the species used in Ecuador were added to the pharmacopoeia within the last 200 years (Fig. 13).

**Figure 14:**
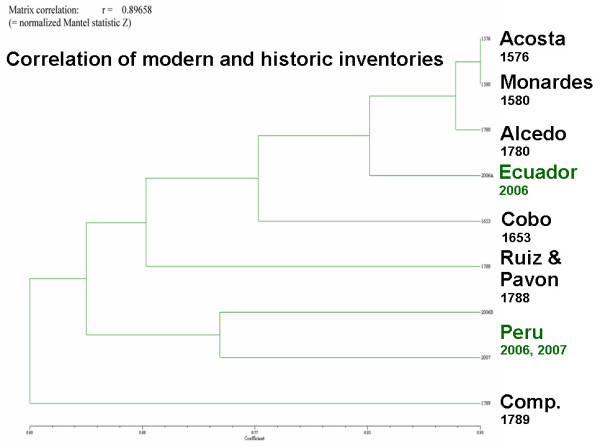
**Matrix correlation of modern medicinal floras to colonial records and botanical inventories**.

A cluster analysis of the colonial and modern plant inventories (Fig. 14) provides a striking explanation for the use differences between Ecuador and Peru, and helps to explain why the plant inventories changed so significantly in the eighteenth century: The dendrogram (Fig. 14) indicates that the current pharmacopoeia of useful flora in Ecuador is most similar to the early colonial flora mentioned in Monardes [12], Acosta [7], Cobo [9,10] and Alcedo [8]. This suggests that the Ecuadorian medicinal flora did not develop much between early and late colonial times. In contrast, the modern Peruvian healing flora is much more similar to later collections. An explanation for this lies in the different treatment of traditional practices in Ecuador and Peru; in Ecuador, traditional medicinal practitioners were immediately persecuted once the colonial administration took hold, while the Peruvian administration was much more tolerant. This also reflects in the establishment of a National Institute for Traditional Medicine in Peru in the 1980s, while traditional medicine was illegal in Ecuador, until a constitutional change in 1998 [66,67,69,70,77]. This meant that Ecuadorian healers had no opportunity to experiment with new species to cure diseases introduced by Europeans, while Peruvian healers were able to explore the rich flora of the region in order to find new remedies. This experimentation also extended to "magical" disease concepts like *Mal Aire, Mal Ojo, Susto*, and *Envidia *that were introduced from Spain during the colonial regime. Peruvian healers developed a vast array of medicinals to treat these conditions, which, to a large extent explains the shift in the medicinal flora between the late 1700s and modern times. Experimentation in Ecuador remained restricted to the treatment of common diseases, while spiritual treatments were outlawed until a constitutional revision in 1998 recognized the right of the population to use traditional medicinal practices.

## Conclusion

Current research indicates that the composition of the local pharmacopoeia has changed since colonial times [1]. However, in Northern Peru the overall number of medicinal plants employed seems to have remained at a comparable level, while plant use in Southern Ecuador has decreased. This indicates that the Northern Peruvian health tradition is still going strong, and that the healers and public are constantly experimenting with new remedies. One example of this is the sudden appearance of Noni (*Morinda citrifolia*) fruits and products in large quantities in plant pharmacies and markets in the region since 2005. This plant was not available before, but is heavily marketed worldwide. Peruvian sellers are clearly reacting to a global market trend and are trying to introduce this new species to their customers. This indicates that local herbalists and herb merchants are carefully watching international health trends to include promising species in their own repertoire. In Southern Ecuador, healers were not able to experiment with new remedies due to persecution and legal restrictions. As a result, the pharmacopoeia in this region remained on an early colonial level, with loss of significant knowledge.

The use of hallucinogens, in particular the *San Pedro *cactus (*Echinopsis pachanoi*), is still a vital component of Andean healing practices, and has been around for millennia [16,62,80-83]. *San Pedro *can often be found in Cupisnique and Moche iconography [2,3]. Five hundred years of suppression of traditional healing practices by Western medicine has not managed to destroy this tradition in Peru. The use of *San Pedro*, together with additives like Angel's Trumpet (*Brugmansia *spp.), Jimsonweed (*Datura ferox*), and tobacco, is still a central part of curing ceremonies in Northern Peru. Healers are in fact experimenting with new hallucinogens, and some northern *curanderos *have started to include decoctions of Ayahuasca (*Banisteriopsis caapi*) in their rituals.

Although not formally acknowledged, Southern Ecuador falls into the Northern Peruvian cultural area. It appears to represent a region where traditional plant knowledge, though important, has declined considerably. Southern Ecuadorian *curanderos *and *parteras *(midwives) having almost entirely abandoned indigenous rituals. In fact, *San Pedro *usage was not mentioned as a mind-altering plant by any healer or midwife interviewed, and was not used in curing ceremonies. Centuries of prohibition have led to a pronounced abandonment of traditional knowledge. This is also reflected in the current study. Many plants used for "magical" purposes in Peru [23] have disappeared from traditional use in Ecuador. The fear of prosecution is still very deeply rooted in the healer community, and most healers interviewed stated that they did not wish to be cited by name. Most healing altars or *mesas *in Southern Ecuador are almost entirely devoid of any "pagan" objects such as seashells, pre-Columbian ceramics, etc. Patients are cleansed by spraying them with holy water and perfumes. In rare cases tobacco juice and extracts of Jimson weed (*Datura ferox*) are used to purify the patients. Southern Ecuadorian *mesas *are also much less elaborate than the *mesas *of Peruvian *curanderos *(Figs. 2 and 3). The incantations used by healers during their curing sessions center on Christian symbolism. References to Andean cosmology are almost entirely absent, and the use of guinea pigs as diagnostic instruments has all but disappeared from the tool kit of these healers.

Interestingly, Peruvian *curanderos *have started to fill this spiritual void in Southern Ecuador. Healers from the Northern Peruvian mountains and coastal plains frequently cross over to Ecuador to offer their services to patients – including increasing numbers of foreigners with a "New Age" orientation – who are not satisfied with the more Westernized approach of Ecuadorian healers. These Peruvian colleagues have much more elaborate plant knowledge, and their *mesas *as well as their incantations follow a more traditional pattern.

The knowledge of medicinal plants is still taught by word of mouth, with no written record. Illustrated identification guides for the medicinal plants of Northern Peru and Southern Ecuador and their uses [67,68] will hopefully help to keep the extensive traditional knowledge of this area alive. However, Traditional Medicine is experiencing increasing demand, especially from a Peruvian perspective, as indicated by the fact that the number of herb vendors, in particular in the markets of Trujillo, has increased in recent years. Also, a wide variety of medicinal plants from Northern Peru can be found in the global market. While this trend might help to maintain traditional practices and to give traditional knowledge the respect it deserves, it poses a serious threat, as signs of over-harvesting of important species are becoming increasingly apparent.

Today the most serious threat to this millennial tradition is the destruction of medicinal plant habitats. Urban sprawl has already greatly altered the coastal plains around Trujillo and Chiclayo. Climatic change is threatening the mountain forest systems that are the source of many medicinal species. Most importantly, the high Andean ecosystems and sacred lagoons where many medicinally active species are found are in danger of being destroyed by large-scale mining activities [91].

## Authors' contributions

Both authors share the contributions to fieldwork, data analysis, and compilation of this manuscript.

## Declaration of competing interests

The authors declare that they have no competing interests.
